# A review on nondiabetic hypoglycemia from various causes: Case series report

**DOI:** 10.1097/MD.0000000000036273

**Published:** 2023-11-24

**Authors:** Lulu Gan, Xuan Zhu, Yue Gao, Mingyao Zhong, Shibo Liao, Gao Huang, Yimin Yan

**Affiliations:** a Department of Endocrinology, Xiaogan Hospital Affiliated to Wuhan University of Science and Technology, The Central Hospital of Xiaogan, Xiaogan, Hubei, China; b Medical College of Wuhan University of Science and Technology, Wuhan, China.

**Keywords:** insulin autoimmune syndrome, insulinoma, nondiabetic hypoglycemia, non-islet cell tumor-induced hypoglycemia

## Abstract

**Rationale:**

Hypoglycemia is common in patients with glucose regulation disorders and related diabetic treatments but is rare in nondiabetic patients. Severe hypoglycemia can cause harm to patients’ cognition, consciousness, central nervous system, cardiovascular and cerebrovascular system, and even death. However, the most fundamental way to control hypoglycemia is to identify the cause and deal with the primary disease. This article introduces 3 cases of nondiabetic hypoglycemia with different causes, aiming to improve our understanding of nondiabetic hypoglycemia and improve the ability of early diagnosis and differential diagnosis.

**Patient concerns:**

Case 1 is a 19-year-old female with a history of recurrent coma, and magnetic resonance imaging and endoscopic ultrasound of the pancreas suggest insulinoma. Case 2 is a 74-year-old male with a history of viral hepatitis, and computerized tomography shows multiple nodules in the liver, which is diagnosed as liver cancer. Case 3 is a 39-year-old female with a history of taking methimazole, who tested positive for insulin antibodies, and was diagnosed with insulin autoimmune syndrome.

**Diagnosis:**

All 3 patients were diagnosed with nondiabetic hypoglycemia, but the causes varied, and included insulinoma, non-islet cell tumor-induced hypoglycemia, and insulin autoimmune syndrome.

**Interventions:**

Case 1 underwent pancreatic tail resection; case 2 refused anti-tumor treatment and received glucose injections for palliative treatment only; and case 3 stopped taking methimazole.

**Outcomes:**

After surgery, the blood sugar in case 1 returned to normal, and the blood sugar in case 2 was maintained at about 6.0 mmol/L. The symptoms of hypoglycemia gradually improved in case 3 after stopping the medication.

**Lessons:**

Non-diabetic hypoglycemia requires further examination to clarify the cause, and the correct differential diagnosis can provide timely and effective treatment, improving the patient’s prognosis.

## 1. Introduction

Hypoglycemia is a syndrome characterized by a blood glucose concentration below 2.8 mmol/L and sympathetic nervous system excitement, which can result from a number of causes. It is common in patients being treated for diabetes.^[1]^ Non-diabetic hypoglycemia can be caused by various reasons, such as hyperinsulinemia, drug factors, critical illness, insulin antagonistic hormone deficiency, congenital carbohydrate metabolism enzymatic deficiency, and idiopathic reactive hypoglycemia.^[2]^ The most common causes of nondiabetic hypoglycemia in clinical practice include insulinoma, non-islet cell tumor-induced hypoglycemia, and insulin autoimmune syndrome (IAS). Here we retrospectively assess 3 rare cases of nondiabetic hypoglycemia admitted to our hospital and compare them with relevant literature. We aim to improve the ability of clinical departments to differentiate nondiabetic hypoglycemia in practice.

## 2. Case reports

### 2.1. Case 1

Patient 1 was a 19-year-old female admitted to the hospital because of symptoms described as an “intermittent coma for 19 days.” The patient had felt tired, drowsy, and had slow cognition. Nineteen days prior to admission, the patient’s roommate attempted to call and wake the patient without success. The patient was sent to the hospital and her fingerstick blood glucose was 1.9 mmol/L. She recovered consciousness after a high-glucose infusion, and the patient was discharged.

The patient’s second hypoglycemic event occurred when the patient was walking and she suddenly became unconsciousness without limb convulsions and began foaming at the mouth. She was sent to the hospital again, and her venous blood glucose was 3.02 mmol/L. Her medical history was unremarkable, and she had no history of special medication. Her physical examination indicated slow reaction time and slow speech but no abnormalities in the nervous system.

Her auxiliary serum assessments indicated the following: hemoglobin: 109.00 g/L (reference value 115–150 g/L); liver and kidney function, electrolytes, thyroid function, coagulation function, hepatitis B markers, C-12 tumor markers: within normal range; plasma cortisol: 14.56 μg/dL (8 am), 5.75 μg/dL (4 pm), 4.32 μg/dL (12 pm) (reference value: 6.20–19.40 μg/dL); plasma adrenocorticotropic hormone: <5 pg/mL (reference value: <46 pg/mL); blood glucose: 1.91 mmol/L (low) (reference value: 3.9–5.8 mmol/L); insulin: 178.4 uIU/mL (high) (reference value: 2.6–20.7 uIU/mL); C-peptide: 2321 pmol/L (high) (reference value: 370–1470 uIU/mL); glycated hemoglobin: 3.60% (low) (reference value: 3.8–6.5%); anti-insulin antibody: negative; serum anti-glutamic acid decarboxylase antibody: negative; insulin autoantibody (IAA): negative; anti-islet cell antibody: negative; and oral glucose tolerance test (OGTT) and insulin release test (IRT): positive (Table 1).

**Table 1:** OGTT and IRT of Cases 1 to 3.

Case		0 min	30 min	60 min	120 min	180 min
1	OGTT (mmol/L)	2.22	7.92	8.62	7.71	0.75
	IRT (uIU/mL)	19.12	107.40	73.56	68.33	7.80
2	OGTT (mmol/L)	4.1	7.8	9.5	11.6	9.1
	IRT (uIU/mL)	13.96	50.36	67.29	92.35	50.68
3	OGTT (mmol/L)	2.00	8.31	11.42	7.51	2.3
	IRT (uIU/mL)	>1000	>1000	>1000	>1000	>1000

IRT = insulin release test, OGTT = oral glucose tolerance test.

**Figure 1.**
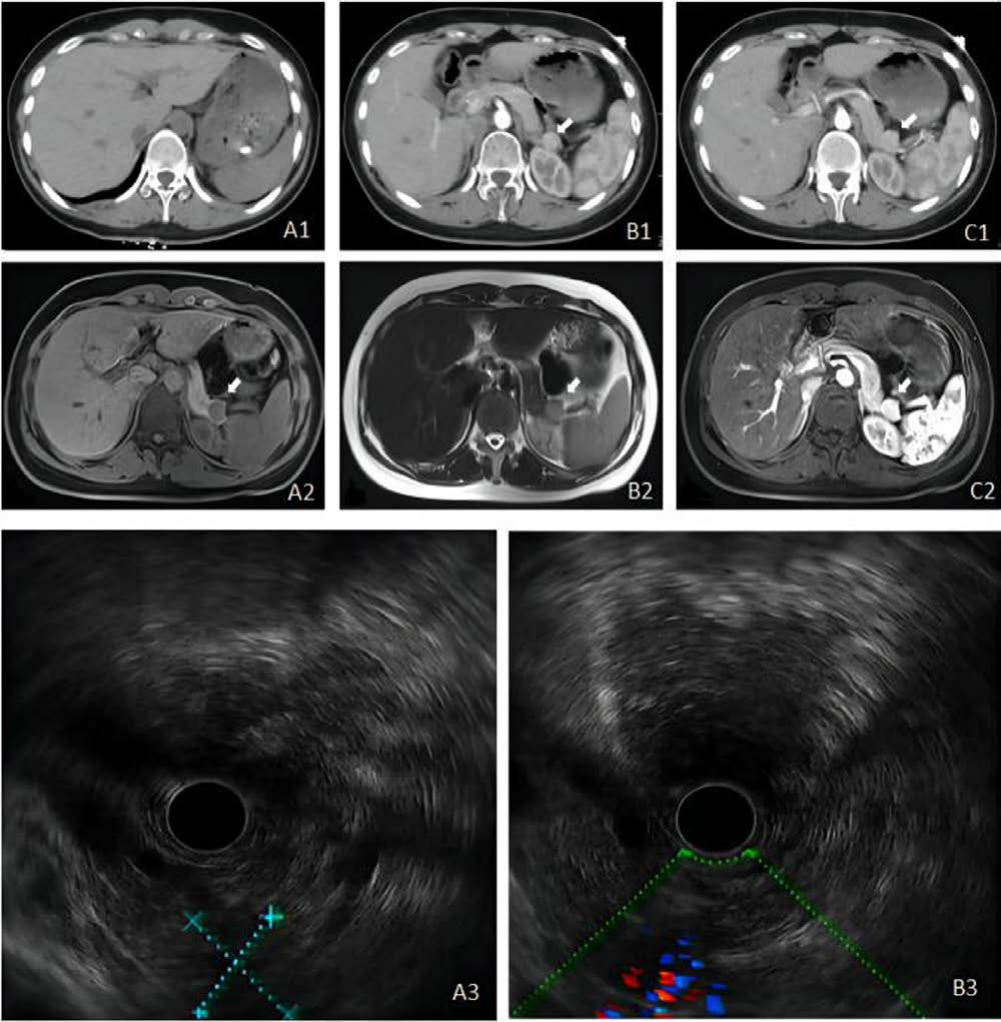
(A1, B1, and C1) Abdominal CT plain scan and enhanced scan of Case 1 indicating suspected lesion in the pancreas. (A2, B2, and C2) Pancreatic MRI of Case 1 showing blood supply of the pancreatic tail, indicating a neoplastic lesion and the high possibility of neuroendocrine tumors. (A3 and B3) Pancreatic abnormalities in endoscopy ultrasound examination of Case 1, including a pancreatic tail lesion indicating the possibility of a neuroendocrine tumor. CT = computerized tomography, MRI = magnetic resonance imaging.

The patient’s imaging results indicated the following: pancreatic computerized tomography (CT) plain + enhanced + three-dimensional: suspected lesion in the pancreas (Fig. 1); pancreatic magnetic resonance imaging (MRI): blood supply of the pancreatic tail was affected, suggesting a neoplastic lesion and the high possibility of neuroendocrine tumors (Fig. 1); electronic gastroscopy ultrasound (US): a pancreatic tail lesion was found, indicating the possibility of a neuroendocrine tumor (Fig. 1).

**Figure 2.**
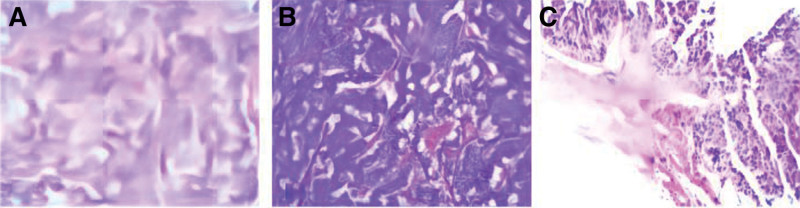
(A and B) Pathology of pancreatic tumor specimens of Case 1. (C) Pathological results of liver puncture biopsy of Case 2.

After all relevant examinations were complete, the patient was diagnosed with insulinoma. The patient was transferred to the surgery department for laparoscopic preservation of the spleen and a pancreatic tail resection. The patient’s postoperative blood sugar was 7.84 mmol/L. The pathological diagnosis was a pancreatic tumor confirmed with a resected specimen (Fig. 2). It was a pancreatic neuroendocrine tumor (G2, tumor size ~1.9 cm × 1.8 cm) with no obvious tumor embolus in the blood vessels. Immunohistochemistry confirmed the following: No. 3 SYN (+), CgA (+), CD56 (+), Ki-67 LI (about 3%), GH (+), plasma adrenocorticotropic hormone (−), AAT (focal+), PCK (+), vimentin (partially +), CD34 (vascular+), D2-40 (vascular+). Three months after surgery, the patient’s blood sugar returned to normal, and she had no further episodes of hypoglycemia.

### 2.2. Case 2

Patient 2 was a 74-year-old male admitted to the hospital because of “intermittent restlessness, sweating for 1 week, and sudden loss of consciousness for 4 hours.” The patient had been feeling hungry for the past week, accompanied by restlessness, sweating, and relief of symptoms after eating. He later developed discomfort below the sternum. At 1 am on the day of hospital admission, the patient felt dizzy. At 7 am, the family found the patient unconscious, unable to respond, with urinary and fecal incontinence, and sent the patient to the emergency department.

Upon admission to the emergency department, the patient’s blood glucose was 2.0 mmol/L, and he recovered consciousness after a 50% glucose 40 mL intravenous injection. Since the onset of the episode, the patient’s mental state has been poor, his physical strength has declined, and his appetite and weight have decreased.

The patient’s physical examination indicated the following: corneal congestion and turbidity, and poor radial reflex. The skin and sclera were yellow, the abdomen was bloated, and there was no tenderness or rebound tenderness. Positive shifting dullness.

The patient’s blood cell analysis indicated: red blood cells: 3.58 × 10^12^/L (low) (reference value: 3.92–5.61 × 10^12^/L); hemoglobin: 101.00 g/L (low) (reference value: 130–175g/L); prothrombin time: 14.3 seconds (high) (reference value: 9–14 g/L); and international standardized ratio: 1.32 (high) (reference value: 0.68–1.3).

His biochemical profile showed the following: aspartate aminotransferase: 56 U/L (high) (reference value: 9–50 U/L); alanine aminotransferase: 135 U/L (high) (reference value: 15–40 U/L); prealbumin: 66.0 mg/L (low) (reference value: 200–400 mg/L); albumin: 35.8 g/L (low) (reference value: 40–50 g/L); albumin/globulin ratio: 1.14 (low) (reference value: 1.2–2.4); direct bilirubin 8.5 μmol/L (high) (reference value: 9–14 g/L); cholinesterase: 3000 U/L (low) (reference value: 5000–12,000 U/L); monoamine oxidase: 14.3 U/L (high) (reference value: 0–12 U/L); α-L-fucosidase: 77.0 U/L (high) (reference value: 5000–12,000 U/L); alkaline phosphatase 187 U/L (high) (reference value: 40–150 U/L); total bile acid: 33.3 μmol/L (high) (reference value: 0–15 μmol/L); γ-glutamyl transpeptidase: 195 U/L (high) (reference value: 10–60 U/L); β2-microglobulin: 2.84 mg/L (high) (reference value: 1.3–2.7 mg/L); blood sugar: 2.22 mmol/L (low) (reference value: 3.89–6.11 mmol/L); retinol-binding protein: 13.4 mg/L (low) (reference value: 25–70 mg/L); potassium: 3.29 mmol/L (low) (reference value: 3.5–5.3 mmol/L); lactate dehydrogenase: 299 U/L (high) (reference value: 120–250 U/L); and alpha-hydroxybutyrate dehydrogenase: 221 U/L (high) (reference value: 72–182 U/L), and OGTT and IRT results are shown in Table 1.

The patient’s inflammatory markers and hormone levels indicated the following: high-sensitivity C-reactive protein: 88.40 mg/L (high) (reference value: 0–3 mg/L); erythrocyte sedimentation rate: 44.0 mm/h (high) (reference value: 0–15 mm/h); serum high-density lipoprotein cholesterol: 0.70 mmol/L (low); and thyroid function, hypertension, and sex hormones: normal. Tests for Hepatitis A, B, and C virus were negative. Tumor markers for liver cancer were as follows: α-fetoprotein: 186.23 ng/mL (high) (reference value: <20 ng/mL); carbohydrate antigen 199: 78.50 IU/mL (high) (reference value: <27 IU/mL); carbohydrate antigen 125: 268.00 IU/mL (high) (reference value: <35 IU/mL); carcinoembryonic antigen: 2.34 ng/mL (high) (reference value: <5 ng/mL).

**Figure 3.**
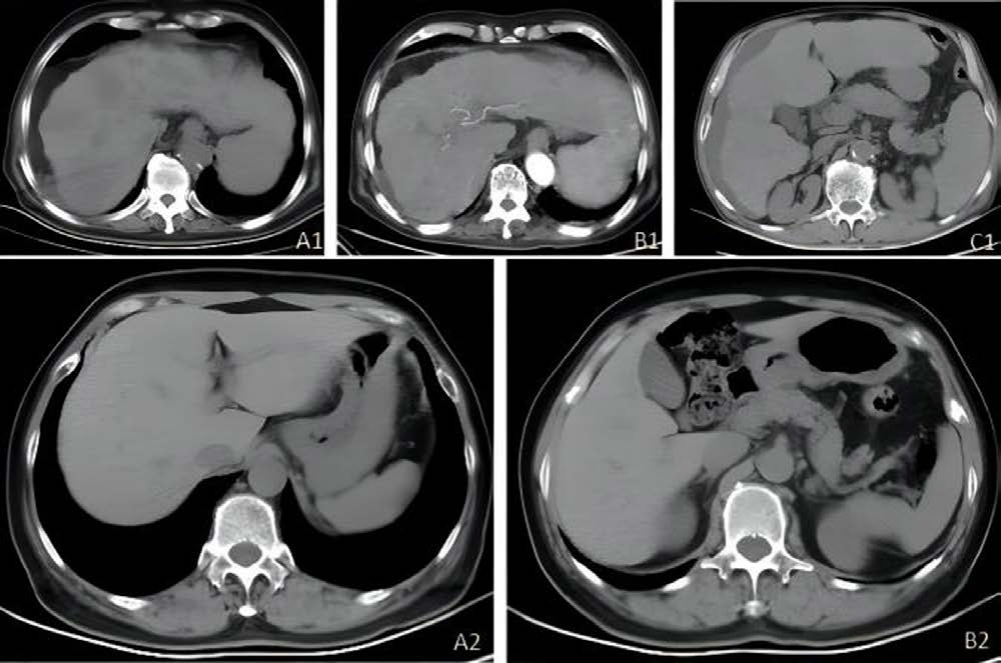
(A1, B1, and C1) Abdominal CT plain scan and enhanced scan of Case 2, indicating multiple liver nodules. (A2 and B2) Abdominal CT plain scan and enhanced scan of Case 3, indicating no significant abnormalities. CT = computerized tomography.

The patient’s abdominal CT showed multiple liver nodules (Fig. 3), and a liver puncture biopsy indicated that the combination of morphology and immunohistochemistry was consistent with hepatocellular carcinoma. Immunohistochemistry indicated the following: No. 2: AFP (+), Arg-1 (-), CD34 (vasculature +), CK19 (+), Glypican-3 (+), Hepatocyte (-), Ki-67 LI (approximately 60%), P53 (wild type) (Fig. 2).

After all assessments were completed, the patient was diagnosed with liver cancer. The patient refused anti-tumor treatment and received glucose injections for palliative treatment only. Subsequent follow-up revealed that the patient had passed away.

### 2.3. Case 3

Patient 3 was a 39-year-old female admitted to hospital due to “fear of heat, excessive sweating for 6 months, and unclear consciousness for 3 days.” Over the past 6 months, the patient had developed fear of heat, excessive sweating, palpitations after activity, accompanied by weight loss and fatigue and had no apparent cause of the symptoms. She was diagnosed with hyperthyroidism in our hospital outpatient department 1 month prior to admission and was treated with 10 mg oral methimazole tablets twice daily. After about 10 days of methimazole treatment, the patient began feeling tired and having intermittent cold sweats. At that time, no treatment was given. Three days prior to admission, the symptoms worsened, and she developed unclear consciousness. She began to speak incoherently, gradually worsening to being unable to recognize people, unclear speech, having limb motions but no seizures, urine and fecal incontinence, or vomiting.

The patient’s physical examination indicated the following: unclear consciousness, delirious state, and equal and round bilateral pupils with dull light response; grade II enlarged bilateral thyroid gland; grade III muscle strength in all 4 limbs; and negative for pathological signs.

The patient’s auxiliary examination indicated the following: biochemistry: blood glucose: 2.3 mmol/L (low) (reference range: 4.16–6.44 mmol/L); bicarbonate: 34.1 mmol/L (high) (reference range: 21–28 mmol/L); anion gap: 4.40 mmol/L (low) (reference range: 8–16 mmol/L); lactate dehydrogenase: 268 U/L (high) (reference range: 115–225 U/L); thyroid function test: FT3: 6.24 pmol/l (reference range: 3.1–6.8 pmol/L); FT4: 18.96 pmol/l (reference range: 12–22 pmol/L); TSH: 0.01 uIU/mL (low) (reference range: 0.27–4.2 uIU/mL); fasting insulin dilution test: 14,802 uIU/mL (high) (reference range: 2.6–24.9 U/L); anti-islet cell antibody: negative; IAA: positive (5.8 KD); anti-glutamic acid decarboxylase antibody: negative; and OGTT and IRT results are shown in Table 1. The patient’s abdominal CT plain scan and enhancement showed no significant abnormalities (Fig. 3).

After completing all examinations, the patient was diagnosed with IAS and hyperthyroidism. Methimazole was immediately discontinued, and the patient was instructed to eat multiple meals. The symptoms of hypoglycemia gradually improved, and a 72-hour dynamic blood glucose monitoring was performed, with blood glucose levels ranging from 2.0 to 6.4 mmol/L, 2.7 to 9.7 mmol/L, and 3.7 to 13 mmol/L. One week later, radioactive iodine (I-131) treatment was performed, and the patient was followed up regularly in the outpatient department. The patient did not develop similar symptoms, and the reexamination of thyroid function after 6 months showed hypothyroidism. She was given 50 □g oral levothyroxine replacement therapy, once daily.

## 3. Discussion and conclusions

Non-diabetic hypoglycemia refers to the condition of low blood sugar in patients without diabetes, which is relatively rare in clinical practice. If a patient presents with the typical Whipple triad (blood glucose ≤ 2.8 mmol/L, hypoglycemic symptoms, improvement of hypoglycemic symptoms after glucose intake), further evaluation of the potential causes of hypoglycemia is necessary. There are currently more articles assessing each of the 3 nondiabetic hypoglycemia types separately than comparing them. Our work here provides 3 clinical cases of the most common nondiabetic hypoglycemia and aims to compare the differences between them, including aspects such as pancreatic function, insulin antibodies, and imaging. We also aim to provide some guidance for clinical workers to distinguish the types of nondiabetic hypoglycemia more easily.

Insulinoma can occur at any age, with a male-to-female ratio of 2:3,^[3]^ while non-insulinoma pancreatogenous hypoglycemia syndrome (NIPH) is more common in the elderly population, and IAS usually occurs in people over 40 years old. There is no significant difference in the sex ratio between IAS and NIPH. In addition, there are 1–4 cases of insulinoma^[4]^ per 1 million people,^[5]^ and the incidence of NIPH is about 1/4 of that of insulinoma. IAS is the third most common cause of nondiabetic hypoglycemia after insulinoma and NIPH, with an incidence of 0.017 cases per 1 million people.^[6]^

Moreover, more than 90% of insulinomas originate from pancreatic β cells, with a probability of 1/3 in the head, body, and tail of the pancreas; and <2% of insulinomas occur outside the pancreas, most commonly on the duodenal wall.^[7]^ NIPH can be divided into interstitial and epithelial tissue tumors according to their tissue of origin, with hepatocellular carcinoma most common, accounting for 23% of NIPH, and 4% to 27% of patients with hepatocellular carcinoma having hypoglycemia.^[8]^

All 3 types of nondiabetic hypoglycemia meet the clinical characteristic criteria of the Whipple triad for hypoglycemic symptoms. Insulinoma and NIPH often occur at night and on an empty stomach. NIPH patients may experience weight loss, pain, palpable masses, sudden coma, and some may also have acromegaly-like changes in their limbs. As the disease progresses, the frequency and severity of hypoglycemia increase. IAS often occurs after meals, and blood sugar can fluctuate.

Furthermore, insulinoma etiology may be due to overexpression of insulin splicing variants and increased translation efficiency in insulinoma.^[9]^ The etiology of hypoglycemia caused by liver cancer in NIPH can be divided into types A and B. Type A usually occurs in the terminal stage and is due to tumor occupation of normal liver, resulting in disorders of glycogen synthesis, decomposition, glycolysis, and gluconeogenesis in the liver, as well as the inability to produce glucose that satisfies tumor consumption.^[8]^ The patient in case 2 is likely to be type A NIPH. Type B occurs in the early stage of liver disease and is due to the excessive secretion of big-insulin-like growth factor II (big-IGF-II) by tumor tissues.^[10]^ Insulin-like growth factor II (IGF-II) has a chemical structure homologous to insulin^[11]^ and produces insulin-like effects by activating insulin receptors. The biological effect of big-IGF-II is 10 times greater than that of IGF-II,^[12]^ which inhibits the glucose output in the liver and increases glucose utilization in skeletal muscles.^[13]^ At the same time, IGF-II can inhibit the release of counter-regulatory hormones, glucagon and growth hormone, exacerbating hypoglycemia. IAS is closely related to HLA-DR4 gene. Susceptible genes found so far include DRB10406, DRB10403, DRB1*0407.^[6]^

Interestingly, treatment with drugs containing thiol groups, such as methimazole, α-lipoic acid, imipenem, and exogenous insulin, most commonly induces the disease. The patient in case 3 was treated with methimazole, a common thiol group-containing drug. Drugs containing thiol groups (-SH) can reduce the disulfide bonds (-S-S-) of insulin, leading to conformational changes in endogenous insulin, triggering an immune response that produces a large amount of IAAs, which combine with insulin to form IAA-insulin complexes.^[14]^ This leads to the loss of biological activity of insulin, causing hyperglycemia. After 3 to 4 hours, the antigen-antibody complex dissociates, releasing too much free insulin, resulting in hypoglycemic symptoms.^[15]^

During both insulinoma and IAS, patients exhibit hypoglycemia and an inappropriate elevation of insulin leading to a qualitative diagnosis. When blood glucose is ≤3.0 mmol/L, insulin ≥3 μU/mL, C-peptide ≥0.6 ng/mL, and proinsulin ≥5 pmol/L, a diagnosis of endogenous hyperinsulinemia can be made. However, if insulin < 100 μU/mL, a diagnosis of insulinoma is more likely. Further, insulin and C-peptide levels of insulinoma rise in parallel, and the insulin release index (insulin/blood glucose) is >0.4, while IAA is negative in most cases of insulinoma.^[16]^ If insulin >100 μU/mL, the diagnosis is most often IAS. However, the total C-peptide concentration measured in IAS patients is much lower than the insulin concentration in the blood, showing a separation of C-peptide and insulin, and insulin autoantibody titer increases in IAS patients. Moreover, NIPH patients often have low levels of insulin, C-peptide, proinsulin, and β-hydroxybutyrate, and some may also have low blood potassium levels, elevated tumor markers, big-IGF-II, and IGF-II/IGF-I > 10, all aiding in the diagnosis of NIPH.^[10]^

To localize the diagnosis, noninvasive diagnosis methods such as US, CT, and MRI are commonly used. However, more than 90% of insulinomas have a diameter of <2 cm, and the sensitivity of US is low (23%–63%,), while CT is 40% to 73%.^[3]^ The diagnosis of insulinoma by MRI is not as accurate as diagnosis by CT, and false-negative results are common. However, the invasive examination method of endoscopic US has an accuracy rate of 96.2%, and the accuracy rate can reach 91% when intraoperative US examination is combined with intraoperative palpation by surgeons. Selective arterial calcium stimulation venous blood sampling and selective pancreatic artery angiography can also be used. The pancreas of IAS patients may also show proliferation and hypertrophy, which is prone to misdiagnosis.

Currently, surgical resection is the preferred treatment for insulinoma, and ethanol ablation can be used for those who cannot tolerate surgery. Chemotherapy with streptozotocin can be used for internal medical treatment, or growth hormone-like substances and molecular targeted drugs can be used as well.^[17]^ However, the most effective treatment for NIPH is still complete surgical resection or liver transplantation. If complete resection is not possible, cell ablation surgery, transarterial chemoembolization (–), percutaneous ethanol injection, and systemic radiation and chemotherapy can be used.^[18]^

Glucocorticoids are the most widely used drug for the treatment of NIPH, which may reduce the effect of big-IGF-II. Octreotide and diazoxide are ineffective for NIPH. The treatment of IAS involves discontinuing the inducing drugs. It usually has self-limited characteristics, and symptoms are resolved within 1 to 3 months after discontinuation. If that is not successful, patients will need to use additional drug treatment, including drugs that limit pancreatic insulin release, such as growth hormone-like substances and diazoxide, as well as immunosuppressants, such as glucocorticoids, azathioprine, and rituximab.^[6]^

In our study, 3 patients with nondiabetic hypoglycemia experienced severe hypoglycemic reactions before seeking medical attention. Case 1, an insulinoma patient, did not experience further hypoglycemic reactions after surgical treatment. Case 3, a patient with IAS complicated by hyperthyroidism, stopped taking methimazole and underwent radioactive iodine treatment, without subsequent hypoglycemic reactions. However, Case 2, a patient with non-islet cell tumor, only received palliative treatment and died during the follow-up period, making it impossible to assess the changes in blood glucose after antitumor treatment.

In summary, hypoglycemia is a common disease in endocrinology with complex and diverse causes. Non-diabetic hypoglycemia can have relatively rare causes, and its differential diagnosis is broad. Treatment should be based on the cause, and a multidisciplinary team can improve prognosis. This work discusses 3 cases of different types of insulinoma, non-insulinoma pancreatogenous hypoglycemia, and IAS, aiming to deepen clinicians’ understanding of nondiabetic hypoglycemia and improve their understanding of the causes of hypoglycemia, as well as aid in their ability to achieve early diagnosis and treatment.

## Acknowledgment

We thank Medjaden Inc. for scientific editing of this manuscript.

## Author contributions

**Conceptualization:** Yimin Yan.

**Data curation:** Lulu Gan, Xuan Zhu, Yue Gao, Mingyao Zhong.

**Formal analysis:** Lulu Gan, Xuan Zhu, Yue Gao, Mingyao Zhong.

**Project administration:** Yimin Yan.

**Writing – original draft:** Lulu Gan, Xuan Zhu.

**Writing – review & editing:** Lulu Gan, Xuan Zhu, Yue Gao, Mingyao Zhong, Shibo Liao, Gao Huang, Yimin Yan.
